# Effectiveness of mHealth Apps for Maternal Health Care Delivery: Systematic Review of Systematic Reviews

**DOI:** 10.2196/49510

**Published:** 2024-05-29

**Authors:** Edward Kwabena Ameyaw, Padmore Adusei Amoah, Obidimma Ezezika

**Affiliations:** 1 Institute of Policy Studies Lingnan University Hong Kong China (Hong Kong); 2 School of Graduate Studies Lingnan University Hong Kong China (Hong Kong); 3 Department of Psychology Lingnan University Hong Kong China (Hong Kong); 4 Global Health & Innovation Lab Faculty of Health Sciences, School of Health Studies Western University London, ON Canada

**Keywords:** mHealth, mobile health, maternal health, telemedicine, technology, health care, newborn, systematic review, database, mHealth impact, mHealth effectiveness, health care applications

## Abstract

**Background:**

Globally, the use of mobile health (mHealth) apps or interventions has increased. Robust synthesis of existing systematic reviews on mHealth apps may offer useful insights to guide maternal health clinicians and policy makers.

**Objective:**

This systematic review aims to assess the effectiveness or impact of mHealth apps on maternal health care delivery globally.

**Methods:**

We systematically searched Scopus, Web of Science (Core Collection), MEDLINE or PubMed, CINAHL, and Cochrane Database of Systematic Reviews using a predeveloped search strategy. The quality of the reviews was independently assessed by 3 reviewers, while study selection was done by 2 independent raters. We presented a narrative synthesis of the findings, highlighting the specific mHealth apps, where they are implemented, and their effectiveness or outcomes toward various maternal conditions.

**Results:**

A total of 2527 documents were retrieved, out of which 16 documents were included in the review. Most mHealth apps were implemented by sending SMS text messages with mobile phones. mHealth interventions were most effective in 5 areas: maternal anxiety and depression, diabetes in pregnancy, gestational weight management, maternal health care use, behavioral modification toward smoking cessation, and controlling substance use during pregnancy. We noted that mHealth interventions for maternal health care are skewed toward high-income countries (13/16, 81%).

**Conclusions:**

The effectiveness of mHealth apps for maternity health care has drawn attention in research and practice recently. The study showed that research on mHealth apps and their use dominate in high-income countries. As a result, it is imperative that low- and middle-income countries intensify their commitment to these apps for maternal health care, in terms of use and research.

**Trial Registration:**

PROSPERO CRD42022365179; https://tinyurl.com/e5yxyx77

## Introduction

The use of mobile health (mHealth) apps has ascended following the proliferation of wearable devices, live audio-visual communication systems, SMS text messaging, and mobile phone app inter alia [1]. mHealth is defined by the World Health Organization (WHO) as a “medical and public health practice supported by mobile devices, such as mobile phones, patient monitoring devices, personal digital assistants, and other wireless devices” [2]. mHealth has been identified as an essential public health tool for efficient health care delivery, especially in situations where the face-to-face model of care cannot be readily provided [3-5]. The grievous repercussions of the COVID-19 outbreak on the ailing health systems of low- and middle-income countries (LMICs) [6,7], and some high-income countries (HICs) [6-8], partly suffice the need to optimize the use of mHealth apps or interventions. A critical aspect of health care greatly impacted by COVID-19 was maternal health care delivery [9-11].

Nonetheless, mHealth could help subside some of the challenges confronting maternal health care delivery. Recent evidence has reverberated that the continuum of maternity health care has received its fair share of mHealth interventions globally, with increasing use and resultant positive outcomes [12-16]. So far, existing mHealth apps for maternal and newborn health care include the Mobile Technology for Community Health (MOTECH), behavioral change tools, and momConnect [17,18]. There are also mHealth apps for general health care delivery such as the Hospital Authority mobile app in Hong Kong [19]. mHealth aids to offset the human resource gap through diverse technologies designed to support treatment adherence, clinical diagnosis, and enhancement [2]. In this study, the terms “mHealth applications” and “mHealth interventions” are alternated. In addition, “newborn healthcare or care” is subsumed under “maternal healthcare or care.”

It is anticipated that there will be approximately 5.6 billion mobile connections by 2025, with most of these being smartphones [20]. A considerable proportion of these mobile connections are earmarked to occur in LMICs, where worse maternal health conditions occur, as mobile connections are more readily accessible in some instances than clean water and electricity [21,22]. This could suggest that LMICs have an increased propensity to maximize the benefits of mHealth to truncate the alarming maternal and newborn morbidity and mortality, which at present is almost 95% of all global maternal deaths [23]. The utility of mHealth interventions can, therefore, be maximized to curtail the gloomy maternal health situation in some parts of the world.

There is a plethora of systematic reviews that have synthesized the relevance of mHealth interventions used in maternal health care [24-29]. For instance, Ambia and Mandela [26] realized that mobile phone–based interventions lead to a statistically significant rise in the uptake of early infant diagnosis of HIV while Bossman et al [29] highlighted the importance of SMS text messages and voice message reminders toward behavioral change among pregnant women by enhancing antenatal care (ANC) and prenatal care attendance, skill birth attendance, and vaccination uptake [29].

Meanwhile, these reviews have not been synthesized on a global scale to draw consistencies and inconsistencies in the evidence to guide maternal health care models and interventions, to maximize gains. A recent systematic review of systematic reviews rather focused on the effectiveness of mHealth on health issues such as diabetes, heart failure symptoms, hypertension, and other health conditions [30]. The prevailing evidence seems to converge that mHealth interventions are impactful and beneficial for maternal health care in both HICs and LMICs [31-34].

So far, no review has synthesized the existing systematic literature reviews to provide aggregate evidence to guide maternal health care practitioners, clinicians, and policy makers. Robust synthesis of existing systematic reviews on mHealth interventions may be useful in guiding clinicians and policy makers with relevant evidence and further pinpointing aspects requiring further evidence to minimize potential risks associated with the existing maternal health care models. Hence, this systematic review of systematic reviews explores the effectiveness or impact of mHealth apps or interventions on maternal health globally.

## Methods

### Overview

This paper is a systematic review of systematic reviews. The review addressed the following research questions: (1) What mHealth apps are used for maternal health care delivery globally? (2) What is the effectiveness of mHealth apps for maternal health care? and (3) What are the barriers and facilitators in the use of mHealth apps for maternal health care delivery?

We conducted this study in line with the updated guidelines for PRISMA (Preferred Reporting Items for Systematic Reviews and Meta-Analyses; Multimedia Appendix 1) statement and the methodological considerations when including existing systematic reviews [35].

### Protocol and Registration

We developed a protocol to guide the conduct of the study. The protocol was registered with the International Prospective Register of Systematic Reviews (PROSPERO).

### Search Strategy and Information Sources

Our search focused on published systematic reviews that focused on specific mHealth apps used in delivering any maternity health care (ie, prenatal, birth, or postnatal) and newborn care, subsumed under maternal care. A newborn refers to a neonate, thus, a child within 28 days of birth, as conceptualized by the WHO [36]. A systematic search for systematic reviews was executed in 5 databases: Scopus, Web of Science (Core Collection), MEDLINE or PubMed, CINAHL, and Cochrane Database of Systematic Reviews, using a predeveloped search strategy (Multimedia Appendix 2). The aforementioned databases were used due to their dominance in biomedical reviews. Searches were done on October 20, 2022. Besides, relevant references of retrieved papers were searched manually to retrieve other relevant papers.

### Eligibility Criteria

This paper included systematic reviews involving primary studies of all study designs (eg, randomized controlled trials [RCTs], pre- and posttest designs, nonrandomized trials, and observational studies). We included reviews focusing on the effectiveness of all available mHealth apps used for maternal and newborn health care delivery globally, for example, service delivery apps. A paper was considered a systematic review if it had these five characteristics: (1) clearly defined aim or research question, (2) eligibility criteria for included studies, (3) appropriate search strategy, (4) appraisal or quality assessment, and (5) analysis or synthesis [37]. Besides, the Population, Intervention, Comparison, and Outcomes framework was applied in determining the eligibility. Thus, the population of interest was pregnant women, women in labor or at birth, postnatal women, and neonates. The intervention of interest was mHealth as defined in the *Introduction* section. Where reported, the comparison was the women or neonates who were not treated with the assistance of any mHealth app, and the outcomes were the reported results that emerged after using mHealth app for any maternity or neonatal condition. Only studies in the English language were considered without year limits. Due to the language competency of authors, only papers published in the English language were considered as English is the working language of the authors. We also excluded papers focusing on nonmaternal health issues or reviews on interventions other than mHealth.

### Study Selection

Reviews were imported to EndNote (Clarivate), and duplicates were removed, after which title and abstract screening were performed by 2 authors (EKA and PAA) using the inclusion and exclusion criteria. This was done while blinding each other’s decision, and all discrepancies were discussed for resolution. Initial screening was on the titles and abstracts retrieved from the search. All suitable papers were retrieved for full-text evaluation. Afterward, we conducted a full-text assessment to determine the eligibility for inclusion. The journal, authorship, or years were not blinded in the process. Reference lists of included papers were searched for additional suitable papers, and we had no reason to contact authors as our results were exhaustive.

### Quality Assessment, Data Extraction, and Synthesis

We extracted data following a standardized extraction form (EKA), after which the data were reviewed by a senior researcher (PAA). Specifically, we extracted the following data: author information and year of publication, setting or context, objective, time range of included papers, databases searched, number of included papers, design of included papers, and whether the meta-analysis was conducted or not (Table 1). In addition, we extracted data on the description of included mHealth apps, the target population, the effectiveness of the mHealth apps, and other relevant outcomes of interest as shown in Table 2. All discrepancies were discussed for resolution. By way of definition, (1) mHealth apps refer to the specific mHealth technique and tools that were used; (2) the target population could either be pregnant women, women in labor or childbirth, postnatal women, and neonates; and (3) effectiveness refers to the outcomes reported after using the reported mHealth technique or intervention. We presented a narrative synthesis of the findings by highlighting the specific mHealth apps, where they are implemented, and their effectiveness or outcomes toward various maternal conditions, such as the impact on maternal health care use. Each of the authors (EKA, PAA, and OE) independently assessed the quality of the reviews using the critical appraisal instrument for systematic reviews and research synthesis by the Joanna Briggs Institute (Multimedia Appendix 3 [38]). This is a 12-point critical appraisal tool for assessing the quality of a systematic review to determine if specific papers could be included or otherwise. Reporting was guided by the Preferred Reporting Items for Overviews of Reviews (PRIOR).

**Table 1:** Summary of included reviews.

Authors	Setting or context	Aim	Literature date	Databases	Included studies, n	Study design of included studies	Meta-analyzed
Bayrampour et al [39]	Sweden, Australia, United Kingdom, Switzerland, and Canada	To examine the effectiveness of eHealth interventions in reducing perinatal anxiety	2014-2017	MEDLINE, CINAHL, Embase, and PsycINFO	5	RCTs^a^	Yes
Chae and Kim [27]	United States, Sweden, Netherlands, Jordan, Norway, Australia, and Singapore	To investigate the effect of internet-based prenatal interventions among pregnant women	2017-2020	PubMed, CINAHL, Cochrane Library, Embase, and ERIC	15	RCTs	Yes
Daly et al [40]	Ireland, United States, and Australia	To determine the effects of mobile app interventions on healthy maternal behavior and perinatal health outcomes	2016	PubMed, Embase, Cochrane Library, CINAHL, WHO Global Health Library, POPLINE, and CABI Global Health	4	RCTs	No
Eberle et al [41]	Unspecified	To assess the clinical effectiveness of technologies for diabetes in pregnancy	2008-2020	MEDLINE, PubMed, Cochrane Library, Embase, CINAHL, and Web of Science Core Collection	22	RCTs, randomized crossover trials, cohort studies, and controlled clinical trials	No
Farzandipour et al [42]	Australia, United States, Norway, China, United Kingdom, and Europe	To investigate the effects and features of phone-based interventions to control gestational weight gain	2011-2016	MEDLINE (via PubMed), Scopus, and Cochrane Central Register of Controlled Trials	12	RCTs and nonrandomized controlled trials	No
Griffiths et al [43]	United States and United Kingdom	To explore whether digital interventions for pregnancy smoking cessation are effective, the impact of intervention platform on smoking cessation, the associations between specific BCTs^b^ delivered in interventions and smoking cessation, and the association between the total number of BCTs delivered and smoking cessation	1997-2017	Academic Search Complete, ASSIA, CINAHL, The Cochrane Library, Embase, MEDLINE, PsycINFO, Scopus, and Web of Science	12	RCTs and quasi-RCTs	Yes
Lau et al [34]	United States, Spain, Italy, and Ireland	To evaluate the efficacy of internet-based self-monitoring interventions in improving maternal and neonatal outcomes	2007-2015	Cochrane Central Register of Controlled Trials, PubMed, Embase, Cumulative Index to Nursing and Allied Health Literature, PsycINFO, Scopus, and ProQuest	9	RCTs and controlled clinical trials	Yes
Lee and Cho [44]	Switzerland, United States, and United Kingdom	To examine the development and delivery of technology-supported interventions for pregnant women and explored the effects of these interventions on the targeted outcomes	2005-2017	MEDLINE, CINAHL, and Scopus	11	RCTs, pilot RCTs, prospective mixed methods one-group pretest or posttest designs, case reports, pilot interventions, and mixed methods studies	No
Ming et al [45]	Italy, Poland, United States, Spain, and Ireland	To determine whether telemedicine solutions offer any advantages relative to standard care for women with diabetes in pregnancy	1996-2015	MEDLINE, Embase, Compendex, and PubMed	7	RCTs	Yes
Oh et al [46]	United States and Netherlands	To assess the effectiveness of digital interventions in preventing alcohol consumption during pregnancy or pregnancy-planning period, and effectiveness of alternative digital intervention platforms (eg, computers, mobiles, and text messaging services)	2012-2018	PubMed, Embase, CINAHL, and Web of Science	6	RCTs	Yes
Overdijkink et al [47]	Netherlands, United States, Australia, United Kingdom, and Japan	To evaluate the feasibility, acceptability, effectiveness of mHealth^c^ lifestyle and medical apps to support health care during pregnancy	2007-2017	Embase, MEDLINE Epub (Ovid), Cochrane Library, Web of Science, and Google Scholar	29	RCTs, pilot studies, prospective or retrospective cohort studies, surveys, and qualitative health care research	Yes
Rahman et al [48]	Kenya, Nigeria, Brazil, India, Ethiopia, Tanzania, and China	To summarize the effect of mHealth interventions on improving the uptake of ANC^d^ visits, skilled birth attendance at the time of delivery, and facility delivery among pregnant women	2012-2018	APA PsycINFO, British Nursing Index, CINAHL Plus, Embase, MEDLINE, POPLINE, PubMed, The Cochrane Library, and Web of Science	9	RCTs and cluster RCTs	Yes
Silang et al [49]	United States and England	To evaluate the effectiveness of eHealth interventions in treating substance use during pregnancy	2009-2018	PsycINFO, Embase, CINAHL, and Cochrane CENTRAL	6	RCTs	Yes
Silang et al [50]	United States, Netherlands, China, Norway, Australia, United Kingdom, Sweden, Switzerland, and Thailand	To determine the effectiveness of eHealth interventions in preventing and treating depression, anxiety, and insomnia during pregnancy. Second, to identify demographic and intervention moderators of effectiveness	2008-2020	PsycINFO, MEDLINE, CINAHL, Embase, and Cochrane	17	RCTs	Yes
Sondaal et al [51]	Low- and middle-income countries (countries unspecified)	To assess the effect of mHealth interventions that support pregnant women during the antenatal, birth and postnatal period	2001-2014	Cochrane Database of Systematic Reviews, PubMed/MEDLINE, Embase, Global Health Library, and POPLINE	27	Intervention and descriptive	No
Wagnew et al [52]	Zanzibar, Thailand, Kenya, South Africa, Ethiopia, and India	To determine the effectiveness of SMS text messages on focused ANC visits and the attendance of skilled birth professionals	2008-2017	Cochrane Review, CINAHL, PsycINFO, PubMed, and Google Scholar	7	RCTs	Yes

^a^RCT: randomized controlled trial.

^b^BCT: behavior change technique.

^c^mHealth: mobile health.

^d^ANC: antenatal care.

**Table 2:** Summary of key outcomes or findings.

Authors	mHealth^a^ app	Target population	Effectiveness or outcome	Other relevant outcomes
Bayrampour et al [39]	eHealth mental health intervention delivered through computer software, via web websites, or mobile apps	Pregnant and postpartum women	Intervention group had significantly lower depression scores postintervention than the control group. Relative to the control group, more women in the intervention group did not meet the diagnostic criteria for depression (63% vs 12%). Compared with the control group, fewer women in the intervention group met the clinical diagnostic criteria for depression at the 12-week assessment (79% vs 18%; *P*=.001). More women in the intervention group recovered on the Edinburgh Postnatal Depression Scale (62% vs 38%; *P*=.08).	Satisfaction with intervention was high with an associated positive perception. 80% of participants in 1 intervention reported enjoying the program and communicating with their coach. Some participants were stressed about not keeping up with the treatment modules.
Chae et al [27]	Prenatal educational interventions delivered through any web, computer, mobile, eHealth, mHealth, telehealth, app, kiosk, or social networking service platform	Pregnant women	Interventions had a small effect on depression and did not have a demonstrable effect on anxiety. Meta-analysis showed an effect on postpartum depression (−0.16, 95% CI −0.26 to −0.05).	Intervention influenced bonding, breastfeeding efficacy, social support, and quality of life. No influence on nutrition, breastfeeding knowledge, parental self-efficacy, insomnia, work, social adjustment, and health status were observed.
Daly et al [40]	Mobile app–based interventions designed to influence maternal knowledge or behavior during pregnancy	Pregnant women	More intervention participants transitioned to a “maintenance stage” of healthy lifestyle behaviors by 28 weeks of gestation, compared with the control participants (52.8% vs 32.7%; *P*=.004). Intervention participants had an increased daily step at 12 weeks (1096, SD 1898 steps), compared with 259 (SD 1604) steps. By 32 weeks gestation, participants using mobile app recorded information more frequently than the control group. Intervention group had a higher proportion of participants with well-controlled asthma than the control group (82% vs 58%; *P*=.03) at 6 months from baseline.	Clinically significant improvement in the asthma-related quality of life among the intervention group compared with usual care at 6 months from baseline.
Eberle et al [41]	Diabetes technologies: mHealth apps and others	Pregnant woman	Overall, the intervention groups showed lower HbA_1c_^b^ values than the control groups. There were significant differences in support of the intervention groups in patient compliance (defined as the ratio between actual and instructed blood glucose measurements × 100).	—^c^
Farzandipour et al [42]	Intervention using telephone, mobile phone, or smartphone on weight gain control or assessed the feasibility and pilot testing this type of interventions	Obese or overweight pregnant	Most interventions had a statistically significant positive impact on the management of weight gain during pregnancy (7/12, 66%). Meanwhile, some had no effect.	—
Griffiths et al [43]	Digital interventions delivered through computer, video or DVD, mobile telephone, or portable handheld device (eg, tablet or iPad)	Pregnant women who smoke cigarette	The majority of studies reported 7-day point-prevalence smoking abstinence toward the end of pregnancy. There was self-reported abstinence at 8 weeks after intervention.	—
Lau et al [34]	Technology-support self-monitoring intervention	Perinatal diabetic women	Maternal outcome: The meta-analysis revealed that the intervention significantly improved HbA_1c_ levels (mean difference −0.12, 95% CI −0.22 to −0.02). The interventions did not significantly improve cesarean delivery rate for overall effect (RR^d^=0.84, 95% CI 0.68-1.05; z=1.55, *P*=.12). The interventions significantly decreased the cesarean delivery rate among the mixed group (RR=0.73, z=2.23, *P*=.03). Neonatal outcome: No significant differences were found between intervention and control groups in the gestational diabetes mellitus group (mean difference=92.21, z=1.47, *P*=.14) and the mixed group (mean difference=–36.42, z=0.59, *P*=.56). The intervention group demonstrated no significant difference in overall effect (RR=1.09, z=0.24, *P*=.81) compared with the control group.	—
Lee et al [44]	Technology-supported intervention	Pregnant women	Intervention was identified to be effective as conventional interventions. Significant benefits in decreasing maternal stress, depression, enhancing pregnancy-related knowledge, lowering perceived barriers to being active, alcohol abstinence, and smoking cessation. Women reported benefits in stress coping, reduction in anxiety, depression and enhanced bonding with babies.	Suggestions for improvements to the intervention programs included embedding a tracking component for users’ progress over time, not limiting the intervention content to that generated by a schedule and allowing for self-directed use, and expansion of the intervention to pregnant women’s significant others.
Ming et al [45]	Modem transmission of blood glucose readings to a central hospital computer, websites accessible to patients and health care professionals, a telephone system that translated blood glucose readings into audio tones to transmit them to a computer database, SMS text message transmissions of blood glucose readings to a central database, and a telemedicine hub located in the women’s home	Pregnant women diagnosed with gestational diabetes mellitus or with preexisting type 1 or type 2 diabetes	Meta-analysis demonstrated a modest but statistically significant improvement in HbA_1c_ associated with the use of telemedicine technology. 7.5% of women had either pregnancy-induced hypertension or preeclampsia and there was no difference in the risk ratio between the telemedicine or control groups. Rates of cesarean section were high in both groups (50% in the telemedicine and 45% in the control). Mean birth weight was 3363 (SD 115) g and 3302 (SD 121) g for telemedicine group and standard care group respectively. Of the 18% of babies treated for hypoglycemia, no differences in intervention and control groups occurred.	One trial reported mothers’ satisfaction among the intervention group. 90% (17/19) of women in the telemedicine group agreed or strongly agreed that they were satisfied with the (intervention) system and would use it again.
Oh et al [46]	Text4Baby and Text4Baby Pilot (text messaging service), CHOICES intervention (Automated internet intervention), electronic screening and brief intervention facilitating self-change, computer-delivered single session brief motivational intervention, and booster session	Pregnant women or women planning pregnancy	All studies showed that digital interventions may decrease the odds of drinking during pregnancy relative to comparison groups. The primary meta-analysis produced a sample-weighted odds ratio of 0.62 (95% CI 0.42-0.91; *P*=.02) in favor of digital interventions decreasing the risk of alcohol consumption during pregnancy when compared with controls. Computer or internet-based interventions (odds ratio 0.59, 95% CI 0.38-0.93) were an effective platform for preventing alcohol consumption.	—
Overdijkink et al [47]	App or text message service during pregnancy	Pregnant women and their partners	10 studies reported positively on acceptability. 4 out of 19 studies evaluating effectiveness showed significant results on weight gain restriction during pregnancy, intake of vegetables and fruits, and smoking cessation.	One study on diabetic treatment reported on acceptability with a positive user satisfaction.
Rahman et al [48]	All types of mHealth interventions focusing on improving perinatal health care use, including skilled birth attendance and facility delivery	Healthy pregnant women	Positive effect of mHealth interventions on improving 4 or more ANC^e^ visit irrespective of the direction of interventions. Only 2-way mHealth interventions were effective in improving the use of skilled birth attendance during delivery but the effects were unclear for 1-way mHealth interventions when compared with standard care. Interventions were effective for facility delivery in settings where fewer pregnant women used facility delivery, however, the effects were unclear in settings where most pregnant women already used facility delivery.	—
Silang et al [49]	eHealth intervention (eg, video therapy sessions, telephone, SMS text messaging, and recorded therapy sessions) intending to reduce substance use	Pregnant women	eHealth interventions significantly reduced substance use in pregnant women compared with controls. Only 1 of the included studies showed a statistically significant benefit of eHealth interventions over the control group.	—
Silang et al [50]	eHealth interventions delivered in an electronic capacity (eg, video therapy sessions, telephone, SMS text messaging, self-help interventions, and recorded therapy sessions)	Pregnant women	During pregnancy, eHealth interventions have small effect sizes for preventing and treating symptoms of anxiety and depression and a moderate effect size for treating symptoms of insomnia. With the exception of intervention type for the outcome of depressive symptoms, where mindfulness interventions outperformed other intervention types, no significant moderators were detected.	—
Sondaal et al [51]	Medical and public health practices supported by mobile phones and tablets, using text, audio, images, video or coded data in the form of SMS text messages, voice SMS messages, applications accessible via GPRS^f^, GPS, third- and fourth-generation mobile telecommunications, and Bluetooth	Women in pregnancy, labor and postnatal care up to 28 days post partum	All studies addressing maternal and neonatal service use showed significant increases. mHealth interventions increased maternal and neonatal service use: increased ANC attendance, facility-service use, skilled attendance at birth, and vaccination rates. The total perinatal mortality rate based on stillbirth and neonatal mortality was 19 per 1000 births in the intervention group compared with 36 per 1000 births in the control group.	Accessibility of mHealth interventions was enhanced by providing information in different local languages, using locally-based software. Weaknesses of mHealth interventions included lack of mobile phone ownership, illiteracy and low accessibility to mobile phones among rural women. Strengths of mHealth interventions on usability included ease of use, flexibility in use and adaptability to the time in pregnancy and development within the local context. Weaknesses of mHealth interventions included uncertainty on whether a text is received and the limited length of messages. Barrier to acceptance was recipient fatigue when too many messages were sent. Privacy may also be compromised when the mobile phone is shared among family members.
Wagnew et al [52]	—	—	Statistically significant associations in experimental group was noted as pregnant women who received text messaging had a 174% increase in focused ANC visits and 82% in skilled birth attendance.	—

^a^mHealth: mobile health.

^b^HbA_1c_: hemoglobin A_1c_.

^c^Not available.

^d^RR: relative ratio.

^e^ANC: antenatal care.

^f^GPRS: general packet radio service.

## Results

### Characteristics of Reviews

**Figure 1:**
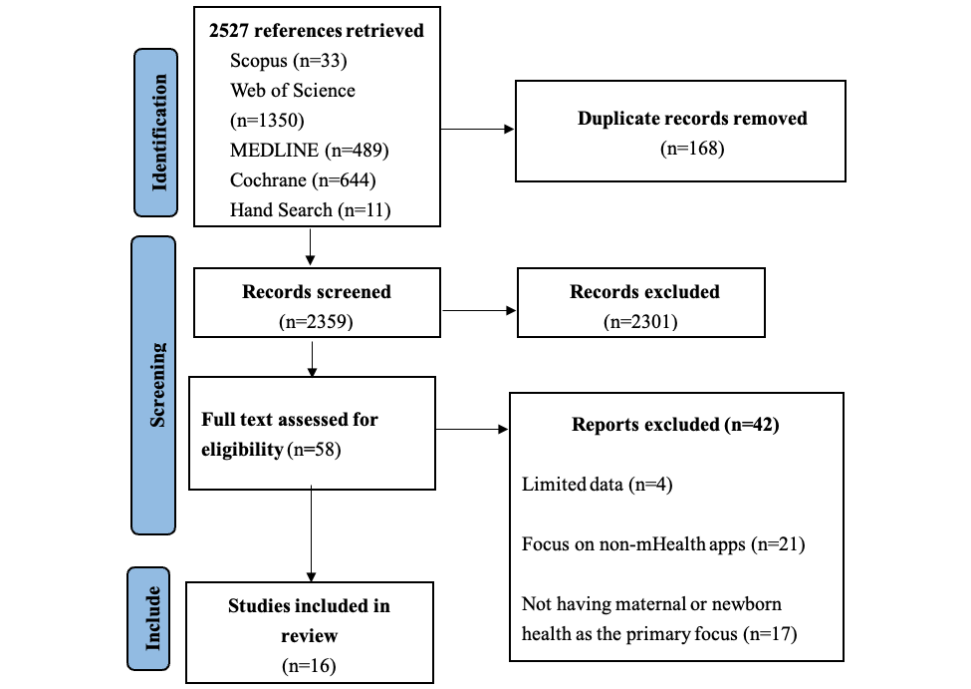
PRISMA (Preferred Reporting Items for Systematic Reviews and Meta-Analyses) 2020 flow diagram for new systematic reviews which included searches of databases and registers only.

Our rigorous systematic search retrieved 2516 papers, with 168 duplicates thereby leaving 2348 papers (Figure 1). An additional 11 papers were identified through cross-referencing and free-hand search. As a result, we screened the titles and abstracts of 2359 papers, and 58 papers were eligible for full-text screening. Of these, 19 papers were deemed eligible for inclusion. However, 3 papers were excluded due to limited data, hence 16 (8.1%) reviews out of 198 studies were included in the final review (Figure 1), as no study was excluded based on quality assessment outcome. Of the 198 studies, 29 (14.6%) studies were included in more than 1 review and they were counted once. These reviews were published between 1996 and 2020 (Table 1), and 11 were meta-analyzed [27,34,39, 43,45-50,52]. In all, 8 included papers on RCT designs only [27,39,40,45,46,49,50,52]; 7 had studies of RCTs and other designs such as controlled clinical trials, cohort studies, non-RCTs, quasi-RCTs, and pilot RCTs [34,41-44,47,48]; and 1 review had no RCT design [51].

Most of the reviews were conducted in HICs (13/16, 81%), predominantly from the United States (n=11) [27,34,40-47, 49,50,52], United Kingdom (n=7) [39,41-43,46, 47,49], Australia (n=6) [27,39,40,42,47,49], the Netherlands (n=4) [27,46,47,50], and Norway (n=2) [41,49]. Only 3 of the reviews were conducted in LMICs (such as Tanzania, Thailand, Kenya, South Africa, Ethiopia, Nigeria, and India) [48,51,52]. In total, 11 (69%) of the 16 reviews focused on pregnant women alone [27,34,40-45,48-50], 2 focused on both pregnant and postpartum women [39,51], 1 was on both pregnant women and women intending to be pregnant [46], and another was focused on pregnant women and their partners [47] (Table 2).

### Characterizing mHealth Interventions and Conditions Supported

Different forms of mHealth interventions are implemented to augment the delivery of maternity health care globally. As illustrated in Table 2, the dominant channels or platforms by which mHealth interventions operate are mobile phones [42,43,51] usually in the form of SMS text messages [45-47,49-51]. Quite a significant proportion (3/16, 19%) of the mHealth interventions are delivered through websites [27,39,45]. With respect to the specific maternal conditions, a substantial proportion of the mHealth interventions were used to assist pregnant or postnatal women in overcoming anxiety and depression [27,39,44,50], assist with diabetes in pregnancy [41,45], and gestational weight [42].

Some were also used to help mitigate smoking cessation [43,47] and alcohol prevention or substance use in pregnancy [44,46,49] or to support ANC attendance and facility delivery [48,51,52]. One review focused on asthma in pregnancy [40]. For instance, in the review by Ming et al [45], they assessed modem transmission of blood glucose readings to a centralized health facility’s telephone system that interpreted blood glucose readings into audio tones by transmitting them to a database. In the case of Farzandipour et al [42], a greater proportion of their studies used the telephone for prenatal weight monitoring, and provision of educational information. The review by Griffiths et al [43] involved interventions delivered by video or DVD, computer (either PC or laptop), mobile phone, or any portable handheld device [43].

### Effectiveness of mHealth Interventions

Considering that different mHealth interventions targeted different conditions in pregnancy and postnatal phases, varied effectiveness outcomes emerged. Meanwhile, all the studies revealed that indeed mHealth interventions are effective for maternal health care, although at varying magnitudes or levels of effectiveness. On depression, there was a uniform agreement among all studies on this condition, demonstrating the extent to which mHealth interventions help mitigate the condition (Table 2). All the reviews on depression highlighted that mHealth interventions were helpful in subsiding depression in pregnancy and childbirth. In the review by Bayrampour et al [39], 4 out of 5 studies revealed positive impacts, with 1 reporting that only 18% of the pregnant and postpartum women met the clinical criteria for depression relative to 79% in the control group, after a 12-week assessment. Similar findings emerged from the review by Lee and Cho [44]. Meanwhile, 2 reviews concluded that mHealth intervention had minimal effect on depression among pregnant women [27,50].

mHealth interventions were reported to be effective in enhancing maternal health care use, as concluded by all the 3 studies focusing on maternal health use. These manifested in ANC enhancement [52], skilled birth attendance at birth leading to a decline in perinatal mortality [51], or both ANC and the use of health facilities for childbirth [48]. Relatedly, the mHealth interventions focusing on alcohol or drinking and smoking showed effectiveness in diverse ways as indicated by all 4 reviews focusing on these [43,44,46,49]. Another area where significant impacts were recorded was obesity or overweight management, especially during pregnancy [42,47] and diabetes management in pregnancy and newborn outcomes [41,45]. For instance, 66% of studies included in the review by Farzandipour et al [42] revealed positive impacts on gestational weight gain. Some studies in the review by Eberle et al [41] also reported marginally higher neonatal birth weight in the intervention group relative to the nonintervention group.

In total, 3 reviews reported that women were satisfied with the mHealth interventions, partly due to the positive user experience and the benefits [39,45,47]. Among the 4 reviews, 1 highlighted dissatisfaction arising from text messaging–based mHealth interventions due to fatigue caused by multiple messages [51]. The same review indicated that the use of mHealth interventions is compromised by privacy issues in instances where the mobile device (eg, mobile phone) is coshared among family members, when women do not own mobile phones, or when there is illiteracy. Two reviews indicated that acceptability and use of mHealth is enhanced by providing information in varied local languages, using locally based software, and when the target is broadened to include women’s significant others [44,51]. Meanwhile, low literacy rate, low accessibility to mobile phones among women in rural locations in LMICs, and uncertainty regarding whether a text is received by the women were some of the identified barriers [51].

## Discussion

### Principal Findings and Comparison With Other Studies

There is sufficient evidence that mHealth apps effectively mitigate diverse conditions that pregnant and postpartum women encounter. All 16 reviews converged that mHealth apps are useful for maternity health conditions. The specific areas where positive impacts manifested include increased use of maternal health care services (ANC, skilled birth, and postnatal care), reduction in perinatal deaths, weight management in pregnancy, alcohol or drinking and smoking mitigation, as well as diabetes management. These conditions represent some of the leading causes of maternal and newborn morbidity and mortality [53].

The positive impact of mHealth interventions on maternity health resonates with prevailing evidence on the implications of mHealth on overall health care such as the review by Marcolino et al [30], which also revealed positive impacts after synthesizing evidence from available systematic reviews. Additionally, consistent evidence has emerged from different parts of the world [54-56]. Continuous and effective use of mHealth interventions in maternal health care can, therefore, constitute a cornerstone strategy for achieving the first and second targets of the third sustainable development goal, which enjoins all countries to attain fewer than 70 maternal deaths per 100,000 live births and reduce neonatal mortality to no more than 12 deaths per 1000 live births by 2030.

Most of the reviews reported evidence from HICs, which is suggestive of limited use of mHealth interventions for maternal health care services in LMICs. As we did not restrict our search to HICs, it is unlikely that evidence from LMICs is missed due to eligibility issues. Hence, we can infer that there is limited use and evidence about mHealth and maternal health services across LMICs. The United States, United Kingdom, and Australia were the dominant locations where mHealth interventions were implemented and assessed. This situation is quite worrying as little evidence exists on the use and effectiveness of mHealth interventions in LMICs, where worse maternal and neonatal morbidities and mortalities occur. Estimates from the World Bank indicate that the United States has a maternal mortality ratio (MMR) of 19, while the United Kingdom and Australia have an MMR of 6 each, as of 2017 [57]. In the same year, African-based LMICs such as South Sudan, Chad, and Sierra Leone recorded MMRs of 1150, 1140, and 1120, respectively [57]. Hence, LMICs with alarming MMRs need to create a more conducive environment for mHealth to thrive. These may include conscious and continuous strengthening of internet connection and electrification, especially in deprived settings.

Several factors could account for the divide in mHealth use for maternal health care between HICs and LMICs. Mobile devices and the internet are essential prerequisites for mHealth activities to thrive. Meanwhile, mobile phone ownership and internet access are quite difficult in most LMICs. For instance, 1 of the 3 reviews that focused on LMICs reported that some women share mobile phones with other family members and this was a setback toward the maximization of the benefits of mHealth interventions [51]. A recent report on mobile phone surveys in 9 LMICs (Colombia, Ghana, India, Indonesia, Kenya, Mozambique, Nigeria, Rwanda, and South Africa) showed that 90% of people in LMICs do not have a decent internet connection [58]. The report also revealed that less than half of the people in LMICs have access to basic internet. The extent to which limited internet access compromises the operation of mHealth in LMICs has been echoed [59-61]. Undeniably, mobile phone access and use in HICs far exceed LMICs [62] and seem to have no relevance without the internet. All these factors might have culminated in the preponderance of mHealth interventions in HICs relative to LMICs.

### Implications for Policy and Future Research Directions

mHealth apps are yielding enormous positive outcomes for maternal well-being. However, the use of mHealth is skewed toward HICs, relative to LMICs where worse maternal conditions emerge from. This is, however, not surprising given the disparity in internet access between LMICs and HICs [63]. Another contributory factor may be the relatively low government expenditure on health care in LMICs, manifesting in limited resource allocation to health care. As of 2019, LMICs averagely spent 5.32% of the gross domestic product on health care as compared with 12.49% average health care expenditure in HICs [64-66]. Hence, an increment in the overall allocation to health care could make it feasible to invest in the most cost-effective mHealth facilities that can offer women the opportunity to benefit from some mHealth interventions that are implementable given the context and acceptability. For instance, simple text message–based mHealth interventions are implementable with relatively minimal resources relative to more advanced options like audio and video conference interventions [67,68].

The use of mHealth interventions typically requires some basic literacy skills [33].

The overall literacy rate of female individuals may, therefore, have to be enhanced for them to benefit from the mHealth interventions. Countries intending to enhance the benefits of mHealth, especially those in LMICs that do not currently have universal compulsory education, ought to give fair consideration to compulsory education or literacy skills training to ensure that all persons attain at least second-cycle education. This could better place female individuals and their partners to use current and future technological innovations not only in health care, but also in all spheres of life. We are not oblivious to the reality that some LMICs already have universal compulsory education systems [69,70]; for those countries, the fortification of such educational arrangements can enhance gains in the long run. More importantly, backing this with a formidable policy framework can guarantee sustainability and effective implementation of mHealth.

For instance, considering internet connectivity challenges in LMICs compared with HICs, text messaging mHealth interventions may be suitable and relatively easily implementable with political commitment backed by a rigorous policy framework. Second, as mHealth interventions require some level of literacy, there is a need for a conscious effort to reduce illiteracy rates. By this, all persons especially women will be well positioned to use mHealth interventions and ascertain the associated benefits. Globally, governments might have to build partnerships or intensify partnerships with the private sector, particularly the telecommunication companies to widen access, as this can offer some leverage for maximizing the use and impact of mHealth interventions for the well-being of women and newborns.

All countries intending to implement mHealth or maximize the benefits of mHealth could implement national mHealth policies that prioritize maternal issues and also invest in internet connectivity and electrification. These are the critical factors that can bridge the gap between HICs and LMICs as well as the rural and urban residents [71,72]. When women and their partners have uninterrupted internet and electricity and a generally suitable environment for mHealth use, minimal advocacy from ANC providers, nongovernmental agencies, and central government can translate into high use and acceptability. By so doing, the alarming MMR battling LMICs could be reduced, with the bridged disparity between HICs and LMICs, leading to a global decline in MMR.

Future research on this subject could focus on how mHealth interventions work out for special populations such as the people with physical disabilities; persons living with HIV in stereotyped settings; teenagers or adolescents; and persons identified with lesbian, gay, bisexual, transgender, and intersex sexual orientations. These populations are sometimes either not comfortable or have truncated access to services meant for the general population [73-76]. Identification of workable mHealth interventions for special populations will augment efforts to attain the global commitment encapsulated in the third sustainable development goal “ensure healthy lives and promote wellbeing for all at all ages” [77].

### Strengths and Limitations

To our knowledge, this is the first systematic review of systematic reviews that has exclusively investigated the effectiveness of mHealth apps or interventions on maternal health globally. Besides, we included all available reviews irrespective of study design and year of publications. This aided in identifying a wide array of mHealth interventions and the myriad of maternity conditions they are used for. Notwithstanding the strengths, the study is not devoid of limitations. First, despite the comprehensive search, we only included reviews written in the English language, which happens to be the working language of the authors. Second, the search in PubMed did not include Medical Subject Headings terms. However, in addition to the database searches, we performed citation searches, specifically forward and backward citation searches, to identify additional reviews that might have been missed in the database search. This was done to identify additional reviews that might have been missed in the database search.

### Conclusions

The effectiveness of mHealth apps in maternal health care is well established in the literature. This manifests in the positive impact toward management and reduction in anxiety and depression, diabetes in pregnancy, and gestational weight. Other aspects include smoking reduction or cessation during pregnancy and enhancement in maternal health care use. Meanwhile, mHealth apps are skewed toward HICs, likely due to the advancement in technology and other resources required to optimize the inherent utility of mHealth apps. Considering that mHealth apps dominate in HICs, LMICs must consider pragmatic approaches to improve their availability and use. Such approaches may include the adoption of workable mHealth policies and political commitment through increment in budget allocation for health care to offer cost-effective mHealth interventions for maternal health care delivery (eg, nonandroid text messages).

## Appendix

Multimedia Appendix 1 PRISMA (Preferred Reporting Items for Systematic Reviews and Meta-Analyses) checklist.

Multimedia Appendix 2 Search Strategy.

Multimedia Appendix 3 Joanna Briggs Institute (JBI) assessment tool.

## Abbreviations

ANC: antenatal care
HIC: high-income country
LMIC: low- and middle-income country
mHealth: mobile health
MMR: maternal mortality ratio
MOTECH: Mobile Technology for Community Health
PRIOR: Preferred Reporting Items for Overviews of Review
PRISMA: Preferred Reporting Items for Systematic Reviews and Meta-Analyses
PROSPERO: Prospective Register of Systematic Review
RCT: randomized controlled trial
WHO: World Health Organization

## Data Availability

All data generated or analyzed during this study are included in this published article (and Multimedia Appendices 1-3).
