# Supplementary material for: Effectiveness of mHealth Apps for Maternal Health Care Delivery: Systematic Review of Systematic Reviews
Source: J Med Internet Res. 2024 May 29;26:e49510. doi: 10.2196/49510 (PMC11170050; doi:10.2196/49510)
Supplement: Multimedia Appendix 2 [file jmir_v26i1e49510_app2.docx]

**Search strategy**

1. **Medline/Pubmed**

| **Mobile Health (mhealth) Application** | |
| --- | --- |
| **#1** | mobile health or mhealth or m–Health or telehealth or ehealth or mobile telemedicine or mobile telehealth care or App or smartphone or ICT or mobile phone or mobile devic*[tiab] or mobile technology or mobile tablet computers or mobile communication or satellite phone or mcare or communication satellite or cell phone or cellular phone or digital health or entreprise digital assistant or personal digital assistant*[tiab] or smartphone* or smart-phone or podcast or apps or pod-cast or mobile applications or short messag*[tiab] or MMS or text messag*[tiab] or short message system or texting or multimedia or multi-media or multi-media tech*[tiab] or multimedia messag*[tiab] or SMS or application or intervention or ios or android or user-computer interface or iPad[tiab] or health application or health techn*or telephone or USSD or palm-top or palmtop or interactive voice response or handheld |
| **Outcome (Mother and Newborn)** | |
| **#2** | maternal or maternal healthcare or maternal health or maternal service or prenatal or pre-natal or prenatal care or prenatal visit* or reproductive health*or reproductive service* or reproductive health serv* or birth or deliver* or labour or antenatal care or preg* or gestation or post-natal or postnatal or postnatal care or postnatal program* or postnatal complications or safe mother* or emergency complication or safe motherhood or perinatal complications or essential preventive maternal health service or obstetric care or newborn or neonatal or neon* or baby or babies or neonatal sepsis or neonatal screening or neonatal nursing |
| **Final string** | |
| **#3** | #1 AND #2 |
| **Limiters** | English, no year limit, global |

tiab, title/abstract

1. **Web of Science (Core Collection)**

| **Mobile Health (mhealth) Application** | |
| --- | --- |
| **#1** | mobile health or mhealth or m–Health or telehealth or ehealth or mobile telemedicine or mobile telehealth care or App or smartphone or ICT or mobile phone or mobile devic*[tiab] or mobile technology or mobile tablet computers or mobile communication or satellite phone or mcare or communication satellite or cell phone or cellular phone or digital health or entreprise digital assistant or personal digital assistant*[tiab] or smartphone* or smart-phone or podcast or apps or pod-cast or mobile applications or short messag*[tiab] or MMS or text messag*[tiab] or short message system or texting or multimedia or multi-media or multi-media tech*[tiab] or multimedia messag*[tiab] or SMS or application or intervention or ios or android or user-computer interface or iPad[tiab] or health application or health techn*or telephone or USSD or palm-top or palmtop or interactive voice response or handheld |
| **Outcome (Mother and Newborn)** | |
| **#2** | maternal or maternal healthcare or maternal health or maternal service or prenatal or pre-natal or prenatal care or prenatal visit* or reproductive health*or reproductive service* or reproductive health serv* or birth or deliver* or labour or antenatal care or preg* or gestation or post-natal or postnatal or postnatal care or postnatal program* or postnatal complications or safe mother* or emergency complication or safe motherhood or perinatal complications or essential preventive maternal health service or obstetric care or newborn or neonatal or neon* or baby or babies or neonatal sepsis or neonatal screening or neonatal nursing |
| **Final string** | |
| **#3** | #1 AND #2 |
| **Limiters** | English, no year limit, global |

tiab, title/abstract

1. **Scopus**

| **Mobile Health (mhealth) Application** | |
| --- | --- |
| **#1** | mobile AND health OR telemedicine OR mobile AND device OR mobile* AND communication OR digital AND health OR smart-phone OR apps OR mobile AND applications OR text* AND message OR short AND message* AND system OR texturing OR multimedia OR multi-media OR multi-media AND tech OR multimedia AND message OR sms OR application OR intervention OR ios OR android OR user-computer AND interface OR ipad OR health AND application OR health OR telephone OR used OR palm-top OR palmtop OR handheld |
| **Outcome (Mother and Newborn)** | |
| **#2** | maternal or maternal healthcare or maternal health or maternal service or prenatal or prenatal care or prenatal visit* or reproductive health*or reproductive service* or birth or deliver* or labour or antenatal care or preg* or gestation or post-natal or postnatal or postnatal care or postnatal program* or postnatal complications or emergency complication or safe motherhood or neonatal or neon* or baby or babies or neonatal sepsis or neonatal screening or neonatal nursing |
| **Final string** | |
| **#3** | #1 AND #2 |
| **Limiters** | English, no year limit, global |

tiab, title/abstract

1. **Cinahl**

| **Mobile Health (mhealth) Application** | |
| --- | --- |
| **#1** | mobile health or mhealth or m–Health or telehealth or ehealth or mobile telemedicine or mobile telehealth care or App or smartphone or ICT or mobile phone or mobile devic*[tiab] or mobile technology or mobile tablet computers or mobile communication or satellite phone or mcare or communication satellite or cell phone or cellular phone or digital health or entreprise digital assistant or personal digital assistant*[tiab] or smartphone* or smart-phone or podcast or apps or pod-cast or mobile applications or short messag*[tiab] or MMS or text messag*[tiab] or short message system or texting or multimedia or multi-media or multi-media tech*[tiab] or multimedia messag*[tiab] or SMS or application or intervention or ios or android or user-computer interface or iPad[tiab] or health application or health techn*or telephone or USSD or palm-top or palmtop or interactive voice response or handheld |
| **Outcome (Mother and Newborn)** | |
| **#2** | maternal or maternal healthcare or maternal health or maternal service or prenatal or pre-natal or prenatal care or prenatal visit* or reproductive health*or reproductive service* or reproductive health serv* or birth or deliver* or labour or antenatal care or preg* or gestation or post-natal or postnatal or postnatal care or postnatal program* or postnatal complications or safe mother* or emergency complication or safe motherhood or perinatal complications or essential preventive maternal health service or obstetric care or newborn or neonatal or neon* or baby or babies or neonatal sepsis or neonatal screening or neonatal nursing |
| **Final string** | |
| **#3** | #1 AND #2 |
| **Limiters** | English, no year limit, global |

tiab, title/abstract

1. **Cochrane Database of Systematic Reviews**

| **Mobile Health (mhealth) Application** | |
| --- | --- |
| **#1** | mobile health or mhealth or m–Health or telehealth or ehealth or mobile telemedicine or mobile telehealth care or App or smartphone or ICT or mobile phone or mobile device or mobile technology or mobile tablet computers or mobile communication or satellite phone or mcare or communication satellite or cell phone or cellular phone or digital health or entreprise digital assistant or personal digital assistant or smartphone or smart-phone or podcast or apps or pod-cast or mobile applications or short message or MMS or or short message system or texting or multimedia or multi-media or SMS or application or intervention or ios or android or user-computer interface or iPad or health application or telephone or USSD or palm-top or palmtop or interactive voice response or handheld |
| **Outcome (Mother and Newborn)** | |
| **#2** | maternal or maternal healthcare or maternal health or maternal service or prenatal or pre-natal or prenatal care or prenatal visit or reproductive health or reproductive service or reproductive health service or birth or deliver or labour or antenatal care or preg or gestation or post-natal or postnatal or postnatal care or postnatal program or postnatal complications or safe mother or emergency complication or safe motherhood or perinatal complications or essential preventive maternal health service or obstetric care or newborn or neonatal or baby or babies or neonatal sepsis or neonatal screening or neonatal nursing |
| **Final string** | |
| **#3** | #1 AND #2 |
| **Limiters** | English, no year limit, global |

tiab, title/abstract
