# Supplementary material for: Effectiveness of mHealth Apps for Maternal Health Care Delivery: Systematic Review of Systematic Reviews
Source: J Med Internet Res. 2024 May 29;26:e49510. doi: 10.2196/49510 (PMC11170050; doi:10.2196/49510)
Supplement: Multimedia Appendix 3 [file jmir_v26i1e49510_app3.docx]

**Table: Critical Appraisal**

| **Article/Review** | **Explicitly stated review question** | **Appropriate inclusion criteria for review question** | **Appropriate search strategy** | **Adequate sources and resources for study search** | **Use of appropriate appraisal criteria** | **Independent critical appraisal by two or more reviewers** | **Presence of methods to minimize errors in data extraction** | **Methods used to combine studies appropriately** | **Assessed likelihood of publication bias** | **Recommendations for policy and/or practice supported by the reported data** | **Appropriate specific directives for new research** | **Overall Appraisal**  **(Yes[include]/No/Unclear=exclude)** |
| --- | --- | --- | --- | --- | --- | --- | --- | --- | --- | --- | --- | --- |
|  |  |  |  |  |  |  |  |  |  |  |  |  |
|  |  |  |  |  |  |  |  |  |  |  |  |  |
|  |  |  |  |  |  |  |  |  |  |  |  |  |
|  |  |  |  |  |  |  |  |  |  |  |  |  |
|  |  |  |  |  |  |  |  |  |  |  |  |  |
|  |  |  |  |  |  |  |  |  |  |  |  |  |
|  |  |  |  |  |  |  |  |  |  |  |  |  |
|  |  |  |  |  |  |  |  |  |  |  |  |  |
|  |  |  |  |  |  |  |  |  |  |  |  |  |
|  |  |  |  |  |  |  |  |  |  |  |  |  |
|  |  |  |  |  |  |  |  |  |  |  |  |  |
|  |  |  |  |  |  |  |  |  |  |  |  |  |
|  |  |  |  |  |  |  |  |  |  |  |  |  |
|  |  |  |  |  |  |  |  |  |  |  |  |  |
|  |  |  |  |  |  |  |  |  |  |  |  |  |
|  |  |  |  |  |  |  |  |  |  |  |  |  |
|  |  |  |  |  |  |  |  |  |  |  |  |  |
|  |  |  |  |  |  |  |  |  |  |  |  |  |
|  |  |  |  |  |  |  |  |  |  |  |  |  |

Yes=Include || No=Exclude || Unclear=Not sure
